# Personal protective equipment for COVID-19 in eye care

**Published:** 2020-09-01

**Authors:** Elanor Watts, Astrid Leck, Victor Hu

**Affiliations:** 1 Doctor and MSc Student: International Centre for Eye Health, London School of Hygiene & Tropical Medicine, London, UK.; 2 Research Fellow and microbiologist: London School of Hygiene & Tropical Medicine, London, UK.; 3 Assistant Clinical Professor: International Centre for Eye Health, London School of Hygiene & Tropical Medicine & Consultant Ophthalmologist, Mid Cheshire NHS Hospitals, UK.

**The main role of personal protective equipment (PPE) within health care settings is to reduce the potential risk of transfer of infectious microorganisms between health care workers and patients. During the pandemic, this is more important than ever.**

**Figure:**
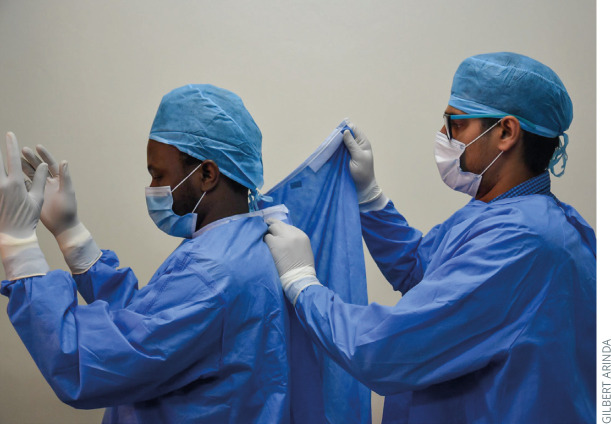
Two health care workers help each other to safely put on PPE. **UGANDA**

Personal protective equipment (PPE) such as gloves, aprons, long-sleeved gowns, eye goggles, face shields (or visors), surgical masks and respirator masks protect health workers and patients. PPE interrupts the chain of infection (Figure 1) by blocking the portals of exit and the portals of entry. This reduces the risk of health workers transmitting the SARS-CoV-2 virus to others, or becoming infected with the SARS-CoV-2 virus themselves.

## Viral transmission

The SARS-CoV-2 virus, responsible for COVID-19, is usually transmitted via small respiratory droplets, produced when an infected person coughs, sneezes, speaks or exhales.^1^ These droplets (bigger than 5 μm in diameter) contain viable viral particles and generally fall from the air within 1 metre of the contagious person.

**Figure 1:**
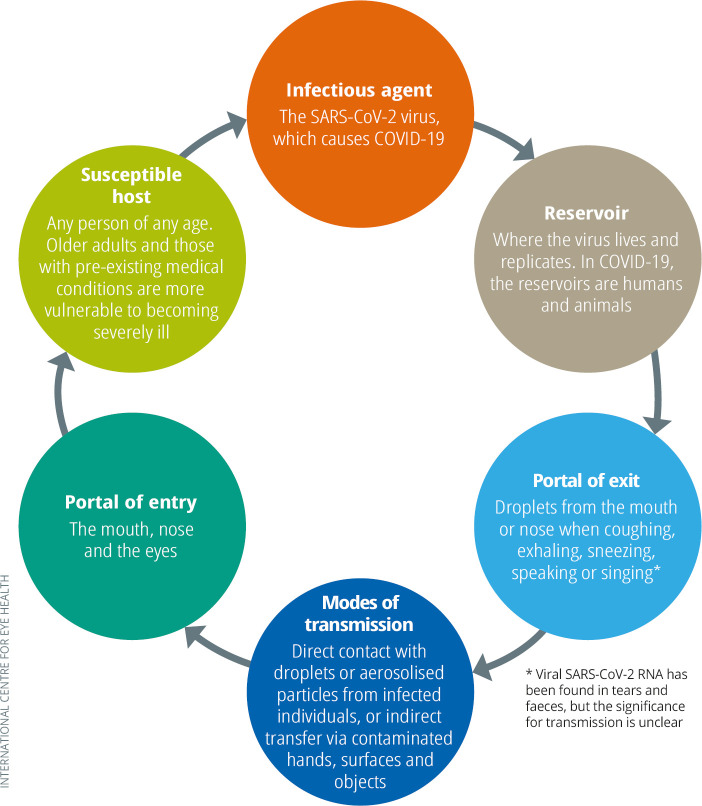
The chain of infection as it applies to COVID-19

**Droplet transmission** takes place when the droplets make direct contact with the conjunctiva of the eyes, or the mucous membranes of the nose and mouth of another person.

**Contact transmission** takes place when someone touches their eyes, nose, or mouth with contaminated hands, e.g., after touching surfaces such as mobile phones, door handles or slit lamps that have already been contaminated by virus-containing droplets (or touched by someone else with contaminated hands). SARS-CoV-2 can survive on smooth surfaces for several days, but is susceptible to standard disinfectant methods.^2^

**Airborne transmission** involves very small droplet particles which can remain in the air for much longer and travel further than droplets before being inhaled.^1^ These particles (or droplet nuclei) are less than 5 μm in diameter and can be produced during aerosol-generating procedures (AGPs) such as endotracheal intubation, upper ENT airway procedures involving suctioning, and non-invasive ventilation (e.g., continuous positive airway pressure). This poses a greater risk to health care workers, so higher levels of PPE are needed, such as a filtering face-piece respirator (e.g., N95 or FFP2 masks) and a fluid-resistant gown. AGPs should also be prioritised when allocating PPE.

There is considerable debate as to whether airborne transmission plays a role in the spread of COVID-19 outside of settings where AGPs are performed. The World Health Organization (WHO) is currently evaluating the role of airborne transmission, and recently said that it “cannot be ruled out” in settings that are crowded, closed and poorly ventilated (see **bit.ly/WHOairbornevideo**). Visit **www.who.int** for the latest updates and guidelines.

**Ophthalmic surgery: which procedures pose a risk of SARS-CoV-2 transmission?**

The SARS-CoV-2 virus can be present in the tear film^3^; however, the relevance of this regarding infection transmission is not yet known. The use of preoperative povidone-iodine as part of standard surgical preparation should inactivate any virus present in the tear film or on the ocular surface.^4^ At present, there is no evidence for the presence of virus in the aqueous or vitreous, but the presence of intraocular virus is theoretically possible.

For phacoemulsification, aerosolisation may occur at the wound edge. However, the aqueous will have been replaced by viscoelastic, and then saline, by the time this happens, so it is unlikely that any aqueous will be present during actual phacoemulsification. Operations such as small-incision and extra-capsular cataract surgery should be low risk. The use of cautery could lead to aerosolisation of virus on the ocular surface; it should be used sparingly, followed by irrigation with saline. The PPE recommended for “Theatres where AGPs not done” in Table 1 should be adequate for such surgery.

Many oculoplastic procedures, especially if involving entry into the nasal cavity or lacrimal drainage system, should be considered high-risk, as should surgery involving general anaesthesia. The PPE recommended for “Performing an aerosol generating procedure (AGP)” in Table 1 should be used.

For up-to-date guidance on the risks of transmission during these and other ophthalmic procedures, refer to the American Academy of Ophthalmology (AAO) guidance^5^ available here: **bit.ly/AAOrisk**.

The use of masks by patients is becoming more widespread in reducing the risk of transmission during a hospital visit.^6–8^ However, there are concerns that a mask may direct the patient’s exhaled breath up and into the surgical field during ophthalmic procedures, which carries a risk of contamination. The mask can also get in the way of cleaning the surgical area and the procedure itself. Once the surgical drape has been properly positioned, it may therefore be advisable to lower the patient mask until surgery is completed.

## What PPE should we use?

Transmission of SARS-CoV-2 can be minimised by:

- Cleaning and disinfecting equipment and surfaces to prevent cross-contamination and spread.
- Washing hands.
- Protecting the eyes,^4^ mouth, nose and clothes by wearing PPE according to national, local or hospital guidelines.

The American Academy of Ophthalmology (AAO) has produced guidance on the risks during eye surgery^5^ (see panel) and the Royal College of Ophthalmologists, UK, has produced guidance on the type of PPE recommended in different situations,^9^ depending on risk (Table 1). Visit **bit.ly/RCOpth** for up-to-date guidance.

**Table 1:** Recommended PPE, as per UK Royal College of Ophthalmologists Guidelines

	Disposable gloves	Disposable plastic apron	Disposable fluid-resistant gown	Fluid-resistant surgical mask	Filtering face piece respirator	Eye/face protection	Slit lamp breath guard
Performing an aerosol-generating procedures (AGPs)	✓ Single use		✓ Single use		✓ Single use	✓ Single use	
High risk acute areas: theatres where AGPs performed, intensive care unit (ITU), high dependency unit (e.g., ophthalmology review of patient in ITU)	✓ Single use	✓ Single use	✓ Sessional use		✓ Sessional use	✓ Sessional use	
Theatres where AGPs not done	✓ Single use	✓ Single use	✓ Single use instead of apron if splashes are likely	✓ Single or sessional use		✓ Single or sessional use	
Working in inpatient area within two metres, e.g., ophthalmology review of ward patients	✓ Single use	✓ Single use		✓ Sessional use		✓ Sessional use	✓ If using a fixed slit lamp
Any outpatient activity (e.g., eye clinic, emergency department)	✓ Single use	✓ Single use		✓ Sessional use		✓ Sessional use	✓

**Single use:** disposal or decontamination of device between each patient/procedure; dispose at end of session

**Sessional use:** dispose at end of session, e.g., at the end of morning clinic or when leaving the care setting

In **low-risk** situations, where there is a risk of droplet and contact transmission only, the advice is to wear disposable gloves, eye protection (goggles or a face shield), a disposable plastic apron and a surgical mask.

In **high-risk situations** (where there is a risk of airborne transmission), the advice is to wear disposable gloves, eye protection, a disposable fluid-resistant gown and a filtering face-piece respirator (N95, FFP2 or FFP3 masks).

### Non-clinical personnel

It is important to recognise that everyone in the health care setting is at potential risk of exposure to SARS-CoV-2, including receptionists, cleaners, pharmacists, administrators, patients and the people who accompany them. Guidelines must be available for all these groups, which includes recommending appropriate PPE and encouraging good hand hygiene.

For example, cleaners should be given full PPE (mask, eye protection, gloves, gown and closed work shoes) and personnel carrying out screening and triage can be protected by enforcing distancing of at least 1 metre between them and others, and by providing a glass or plastic screen as a barrier.

The World Health Organisation (WHO) provides excellent guidance on PPE for everyone working in the health care setting.^10^ **bit.ly/PPEguideCOV19**

## Face masks

**Never** wear a mask just over your mouth and not your nose, as this exposes the mucous membranes of the nose and puts you at risk of inhaling virus-containing droplets.

**Figure 2:**
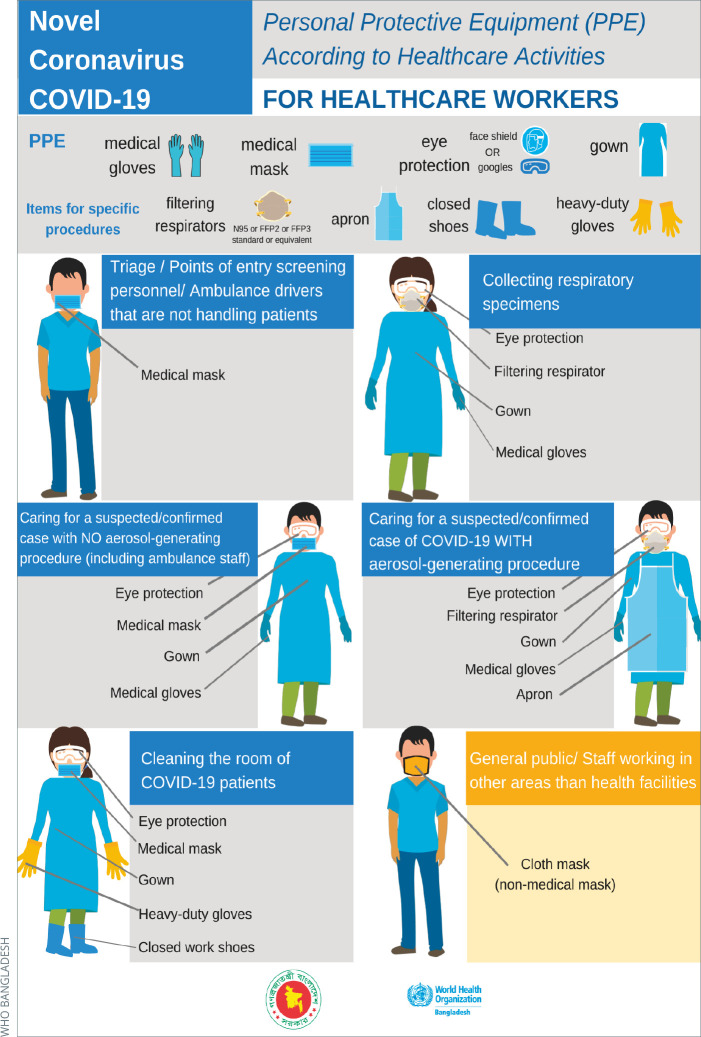
Posters can provide useful reminders about the personal protective equipment (PPE) needed for different activities. **Note:** heavy-duty gloves must be decontaminated or changed between clinical environments, else they can be a source of contamination. Source: **bit.ly/PPEposter**

Different types of face masks are available, with varying levels of protection:

- **Fabric masks** are home-made masks, bandanas or scarves which cover the nose and mouth. The level of protection they offer the wearer is uncertain, and depends on the fabric and the fit.^11^ The purpose of fabric masks is to prevent the wearer from spreading viral particles to others when coughing, sneezing, talking or breathing out, particularly on public transport. Wash fabric masks after each use to prevent them from acting as a fomite: a contaminated surface or object that can spread the virus to others.
- **Surgical masks** should be fluid-resistant to splash or spray of bodily fluids. This type of mask provides some protection against droplet transmission, but the wearer can still breathe unfiltered air around the edge of the mask. The main role of these, and fabric masks, is to reduce transmission of infectious droplets from the wearer to other people. They are suitable for areas with a **low risk** of exposure.
- **Filtering face piece (FFP) respirators.** These form a tight seal around the edge of the mask so all air passes through the filter. This type of mask is designed to protect the wearer and the people they come into contact with from infectious droplets. FFP2 or N95 masks (European and US terminology respectively) filter at least 94–95% of particles bigger than 0.3 µm in diameter. FFP3 or N99 masks filter at least 99% of particles bigger than 0.3 µm. These both meet the WHO criteria for SARS-CoV-2 and can be used in **high-risk** settings and procedures. The only reliable way to distinguish between different FFP masks is to read what is printed on them. FFP1 masks are not sufficient to protect against COVID-19.

Some countries are recommending that patients wear either surgical masks or fabric masks during their clinic visits and/or when outside their home.^6–8^

Exactly when masks are changed will depend on local shift patterns, the frequency of breaks, and PPE supply. FFP respirators can normally only be worn for a relatively short period as the filter fills up after prolonged wear. This means that breathing becomes more difficult for the wearer and the effectiveness of the filter is less certain. Check the manufacturer’s instructions for each specific mask. Many FFP3 respirators should be disposed of **after a maximum of 8 hours**.

## FFP respirators: a good fit is essential

FFP respirators should be fit-tested to ensure an effective seal; if the seal is broken, air and droplets can enter around the edge of the mask. Formal fit-testing involves a health worker wearing the mask and performing different movements and breathing exercises while a strong, bitter substance is sprayed close to their face inside a hood; if they can taste the substance without the mask, but not with it, it fits. If this means of testing is not available, visually inspect that the mask fits snugly to the contours of the face. Observe the wearer breathing in and out to check that the movement in the shape of the mask is consistent with the breathing pattern, i.e., that the front of the mask depresses as they inhale and re-shapes as they exhale. Adjust the general fit and nose-pinching strip accordingly. One mask may not effectively fit all face shapes; ideally, each hospital should have multiple types available to increase the chance of finding one for everyone.

**Figure 3:**
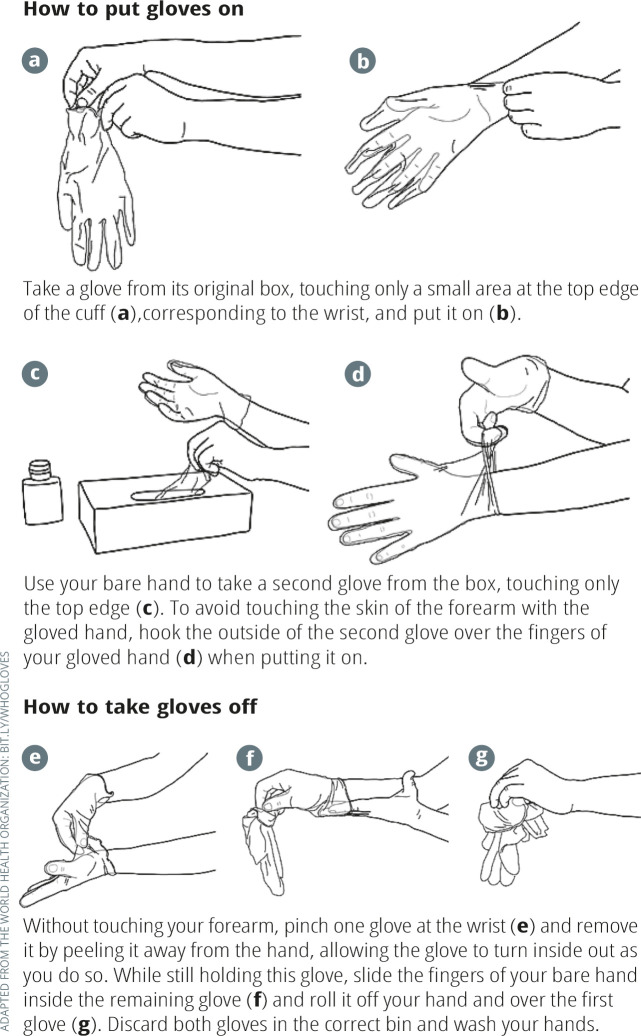
WHO guidance on putting on and taking off gloves

**Figure 4:**
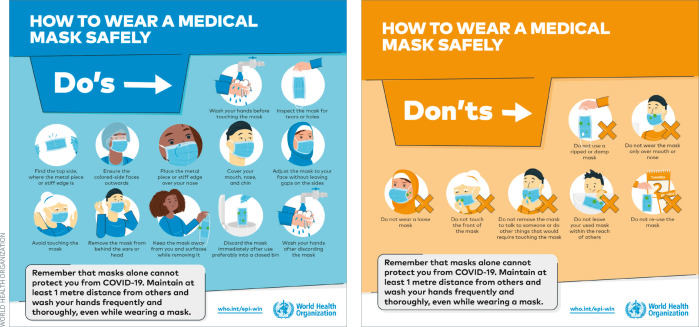
World Health Organization guidance on how to wear a mask safely.

### Facial hair

A good seal is impossible if there is facial hair (including stubble) under the edge of the mask. Some styles of beard or moustache are compatible with a good fit if they do not cross the edge of the mask. If, for cultural reasons, a person cannot remove facial hair that crosses the edge of the mask, it is recommended that their duties are temporarily reassigned. If this is not possible, an alternative option would be to wear a full head respirator.

## Gloves

Use of gloves is indicated during all patient-care activities that may involve exposure to blood and all other body fluids (including contact with mucous membrane and non-intact skin), during contact precautions and outbreak situations.

COVID-19 is an outbreak situation, so glove use is advised for all patient care activities, even in situations ordinarily considered ‘very low risk’ and for which gloves would not usually be indicated.

### Gloves do not provide complete protection

Gloves are only effective when used appropriately (Figure 3) and in combination with good hand hygiene before use and after removal. Prolonged use without adequate hand hygiene may contribute to infection transmission.

It is vital to remove and replace gloves between each patient. Medical gloves are single-use items; decontamination and reprocessing are not recommended and should be avoided, even where glove supply is limited, because there is no standardised, validated and affordable procedure for safe glove reprocessing.

Thick rubber gloves, such as those used when cleaning, should be disinfected between clinical spaces.^12^ For the latest guidance, visit **bit.ly/WHOgloves**

## Eye protection

In many settings, health care workers are encouraged to wear eye protection (goggles or visors/face shields) when in close contact with patients. Eye protection can be re-used.^13^ After each session, clean goggles and face shields using detergent, then using hospital disinfectant. Finish by wiping with water or 70% ethanol to remove any residues.

## Donning and doffing PPE

Just as important as which PPE is worn, is how to put on (don) and take off (doff) PPE safely.

### Donning

**Figure 5:**
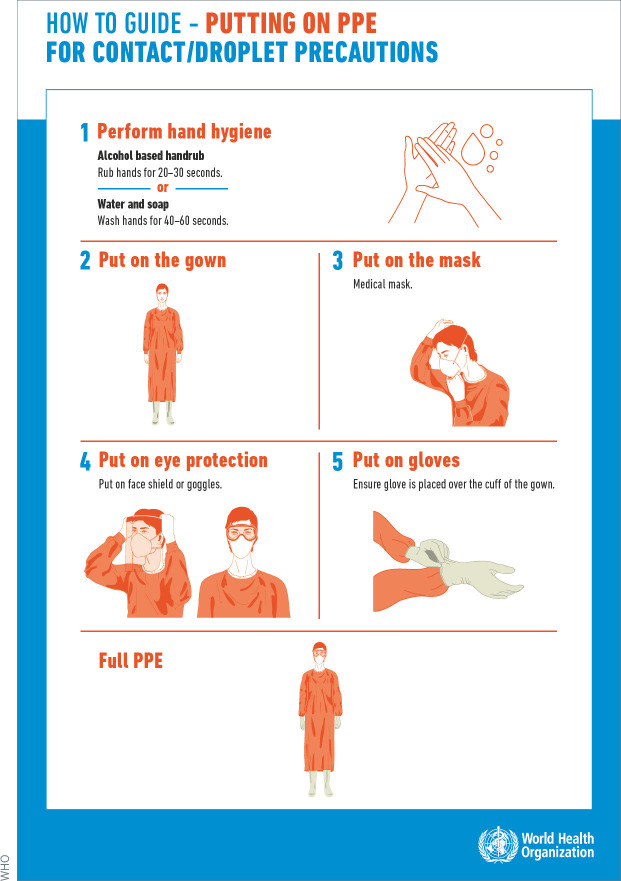
How to don PPE for contact and droplet precautions

Figure 5 shows the order suggested by WHO for donning PPE to avoid contact or droplet transmission:

1. Perform hand hygiene
2. Put on the gown (or apron, if fluid-resistant gowns are not available)
3. Put on the surgical or respirator mask
4. Put on eye protection (goggles or visor/face shield)
5. Put on gloves. Ensure the gloves are placed over the cuff of the gloves.

### Doffing

**Figure 6:**
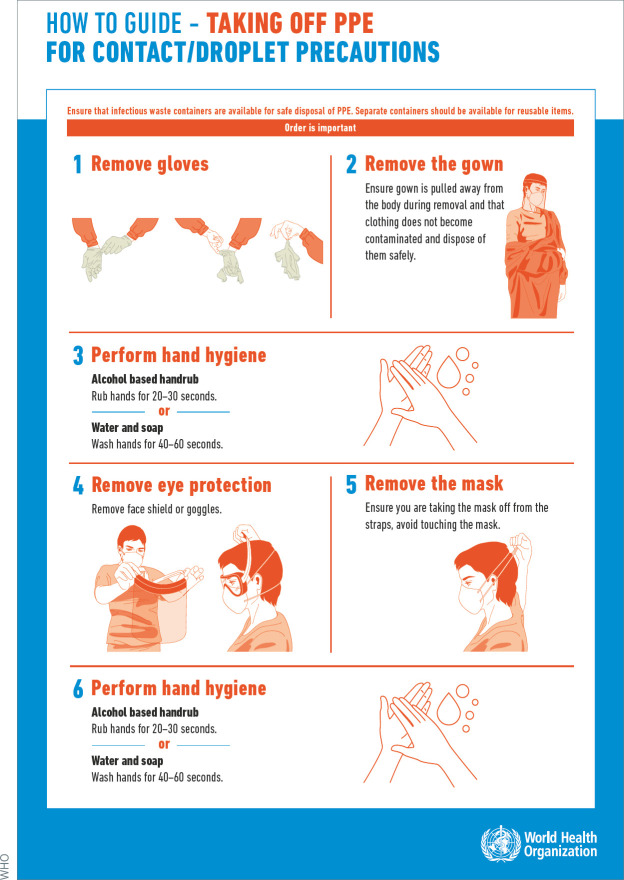
How to don PPE for contact and droplet precautions

Figure 6 shows the order suggested by WHO for taking off PPE worn for contact and droplet precautions.

1. Remove gloves. Avoid touching the outside of the glove. Instead, start at the cuff and peel the glove off, so it is inside-out when you are finished.
2. Remove the gown (or apron, if fluid-resistant gowns are not available)
3. Perform hand hygiene
4. Remove eye protection (goggles or face shield)
5. Remove the mask (or respirator). Ensure that you touch only the straps, not the mask itself.
6. Perform hand hygiene.

**Note:** Hand hygiene must be performed twice: once after removing the gloves and gown, and once when everything else has been removed.

Pay extra attention to avoid contaminating yourself when removing PPE at the end of a session, e.g., by touching the outside of a face visor and then touching your face. If your hands become contaminated at any point during putting on or taking off PPE, wash them immediately. Always wash hands immediately after taking off all PPE. In a high-risk setting, where there is a risk of airborne transmission, a PPE ‘buddy’ should help with putting on PPE, for example, by tying the back of your gown.

Put used PPE in the correct waste or laundry collection receptacle after removal to ensure safe decontamination and disposal.
