# Supplementary material for: Personal protective equipment for COVID-19 in eye care
Source: Community Eye Health. 2020 Sep 1;33(109):28–33. (PMC7677808)
Supplement: Related texts

# Brief report: Making PPE work in your setting

**Published:** 2020

Depending on the local setting and supplies, choose the best available combinations of PPE for both low- and high-risk exposure and develop a strategy or protocol for decontaminating and re-using specific PPE items if needed. All relevant staff members must be made aware of the chosen strategy and taught how to carry it out appropriately. Using the best PPE available in each setting is extremely important in reducing COVID-19 transmission.

Wearing PPE, particularly face masks, can create an additional barrier in patient care, as patients may find it difficult to engage with health care workers if they cannot see their faces. It can also become much more difficult to hear people when they are wearing masks. To assist communication between health care workers, some hospitals are using hand signals to indicate ‘up’, ‘down’, ‘good’, ‘there’s a problem’, etc. across a noisy ward. It is worth considering implementing this in your unit; if you do, make sure that all staff members know what the different signals mean.

Wearing full PPE can be very uncomfortable, especially on hot days, and staff members should look out for each other and be wary if they start to feel faint and remember to take regular breaks. Hydrate before donning PPE in warm climates or warm health care environments.

As described, the reality of using PPE in a clinical setting can be very challenging, including changing equipment between patients and avoiding cross-contamination, especially if there are shortages of proper equipment. It is important to use PPE along with other infection control guidelines such as proper hand washing and waste disposal. We hope that applying these principles, in a common-sense manner, will help to keep health workers and patients safe.

**“Wearing full PPE can be very uncomfortable, especially on hot days, and staff members should look out for each other and be wary if they start to feel faint and remember to take regular breaks.”**

**What if we’re running out of PPE?**

There has been a global shortage of PPE during this pandemic. Many countries and districts have had to modify guidance depending on PPE availability. WHO and the Center for Disease Control (CDC) in the United States have offered helpful guidance on this.^10^,^13^,^14^

Good management of PPE supplies is essential to prevent shortages. This involves forecasting need and monitoring use and distribution. It is important to emphasise that, where possible, PPE of verified quality should always be used, via countries’ official registered sources.^15^ Where alternative sources are required, we must ensure that new supply chains, which may have been developed very quickly, do not involve the exploitation of labourers.

When PPE is in short supply, follow the measures set out in your local or national guidelines. Practices that would be unacceptable in ‘normal’ circumstances have had to be considered due to current extenuating circumstances resulting from this pandemic.

Some PPE can be disinfected and re-used. These include goggles, face shields, fabric gowns or scrubs that can be washed and re-used.

In the current exceptional pandemic crisis, reprocessing of disposable PPE is an evolving area where research and development is ongoing and urgently needed. Reuse of any item without a reprocessing/decontamination process is considered inadequate and unsafe. Normally, cleaning before disinfection and sterilisation is required for any reprocessing methods, which is not possible for masks and respirators.

Methods for reprocessing masks or respirators are not well standardised or established but possible means of decontamination are under investigation for extreme shortages.^16^

- **Prioritise who gets which PPE**, e.g., prioritise face masks for health care providers rather than patients, or prioritise them for use only in close contact and while carrying out care activities which involve splash or spray. Prioritise glove use for healthcare workers engaged with high risk procedures or patients. If glove supplies are extremely limited, hand hygiene alone may suffice when performing very low risk procedures for which gloves are not normally indicated, for e.g., taking a patient’s blood pressure, temperature or pulse – refer to WHO guidelines.^12^
- **Extend the use of PPE** for longer than is normally recommended. E.g., for FFP3 respirators, sessional use can be considered.

**NOTE:** Because advice is regularly being supplemented and revised, please refer to the WHO, RCOphth and AAO websites mentioned in this article for the most up-to-date recommendations.

## References

[1] World Health Organization. Modes of transmission of virus causing COVID-19: implications for IPC precaution recommendations. WHO 2020.

[2] van DoremalenNBushmakerTMorrisDH et al. Aerosol and surface stability of SARS- CoV-2 as compared with SARS-CoV-1. N Engl J Med 2020; 382:1564-1567. doi:10.1056/NEJMc2004973.3218240910.1056/NEJMc2004973PMC7121658

[3] XiaJTongJLiuMShenYGuoD. Evaluation of coronavirus in tears and conjunctival secretions of patients with SARS-CoV-2 infection. J Med Virol 2020; 92(6):589-564.3210087610.1002/jmv.25725PMC7228294

[4] KampfGTodtDPfaenderSSteinmannE. Persistence of coronaviruses on inanimate surfaces and their inactivation with biocidal agents. J Hosp Infect 2020;104(3):246-51.3203599710.1016/j.jhin.2020.01.022PMC7132493

[5] ChodoshJHollandGNYehS. Special considerations for ophthalmic surgery during the COVID-19 pandemic. AAO 2020. Available at: **bit.ly/AAOrisk**

[6] LaiTHTTangEWHChauSKYFungKSCLiKKW. Stepping up infection control measures in ophthalmology during the novel coronavirus outbreak: an experience from Hong Kong. Graefes Arch Clin Exp Ophthalmol 2020; 258(5):1049-55.3212400010.1007/s00417-020-04641-8PMC7087729

[7] Italian Ophthalmological Society. Directions for the protection of patients, ophthalmologists, health and administrative personnel. IOS 2020.

[8] Spanish Society of Ophthalmology. Recommendations for the care of ophthalmology patients in relation to the COVID-19 emergency. SSO 2020.

[9] Royal College of Ophthalmologists. PPE and staff protection requirements for ophthalmology. RCOphth 2020.

[10] World Health Organization. Rational use of personal protective equipment for coronavirus disease (COVID-19) and considerations during extreme shortages. WHO 2020. Available at: **bit.ly/PPEguideCOV19**

[11] DaviesAThompsonKAGiriKKafatosGWalkerJBennettA. Testing the efficacy of homemade masks: would they protect in an influenza pandemic? Disaster Med Public Health Prep 2013; 7(4):413-8.2422952610.1017/dmp.2013.43PMC7108646

[12] World Health Organization. Glove use information leaflet. WHO 2009. **bit.ly/WHOgloves**

[13] Centers for Disease Control and Prevention. Strategies for optimizing the supply of eye protection. CDC 2020.

[14] Centers for Disease Control and Prevention. Strategies to optimize the supply of PPE and equipment. CDC 2020. Available at: **bit.ly/CDCguidePPE**

[15] MariottiSCiezaAHollandP. Eye Health and COVID-19 – A webinar. Online: IAPB 2020.

[16] Centers for Disease Control and Prevention. Decontamination and reuse of filtering facepiece respirators. CDC 2020.
